# Anti-Multiple Myeloma Potential of Secondary Metabolites from *Hibiscus sabdariffa*

**DOI:** 10.3390/molecules24132500

**Published:** 2019-07-09

**Authors:** Alessio Malacrida, Valeria Cavalloro, Emanuela Martino, Arianna Cassetti, Gabriella Nicolini, Roberta Rigolio, Guido Cavaletti, Barbara Mannucci, Francesca Vasile, Marcello Di Giacomo, Simona Collina, Mariarosaria Miloso

**Affiliations:** 1 School of Medicine and Surgery, University of Milan-Bicocca, 20900 Monza, Italy; 2 Experimental Neurology Unit, University of Milano-Bicocca, 20900 Monza, Italy; 3 Department of Earth and Environmental Sciences, University of Pavia, 27100 Pavia, Italy; 4 CREA, Research Centre for Vegetable and Ornamental Crops, 18038 Sanremo, Italy; 5 Center of Large Equipment, University of Pavia, 27100 Pavia, Italy; 6 Department of Chemistry, University of Milan, 20133 Milano, Italy; 7 Department of Drug Sciences, University of Pavia, 27100 Pavia, Italy

**Keywords:** Multiple myeloma, *Hibiscus sabdariffa*, nature-aided drug discovery, bioguided assay fractionation, Hib-ester, Hib-carbaldehyde

## Abstract

Multiple myeloma (MM) belongs to hematological cancers and its incidence is increasing worldwide. Despite recent advances in its therapy, MM still causes many deaths every year. In fact, current therapies sometimes fail and are associated with severe adverse effects, including neurotoxicity. As a part of our ongoing efforts to discover new potential therapies against MM, we prepared *Hibiscus sabdariffa* extracts obtained by a microwave-assisted solvent extraction and investigate their activity by in vitro assays on the RPMI-8226 cell line. The bioguided fractionation of the crude ethanolic extract allowed the identification of HsFC as the most effective extract. We assessed cell viability (MTT and Tripan blue test), cell migration (Boyden chamber assay), and neurotoxicity (DRG neurotoxicity assay). The promising results prompted us to further fractionate HsFC and we obtained two molecules effective against RPMI-8226 cells without neurotoxic effects at their active concentrations. Moreover, both compounds are able to significantly reduce cell migration.

## 1. Introduction

Multiple myeloma (MM) is a plasma cell neoplastic disorder causing severe bone pain and bone fractures, hypercalcemia, anemia, and kidney damage. This disease is characterized by clonal proliferation of malignant plasma cells in bone marrow and by overproduction of monoclonal protein (M protein) [1]. MM represents 1% of all cancer diseases and 10% of hematological cancers and its incidence is highly variable among age, sex, and countries [2]. MM is prevalent among the elderly and the male sex and the most affected countries are the industrialized regions of Australia, Europe, and North America. MM is still considered an incurable disease causing 110,000 deaths every year worldwide. To date, different therapeutic options are available and over the last decade, the five-year relative survival of patients raised approximately to 56% [3]. Nevertheless, the high heterogeneity of MM leads to numerous cases of refractory/relapsing MM. Moreover, since bone marrow is the ideal support for homing and progression of MM cells [4], 70% of patients presents bones metastasis at diagnosis and the percentage further increases to 90% during the development of the disease. These patients suffer from osteolytic lesions caused by osteoblast differentiation suppression and inhibition of bone matrix deposition [5]. Therefore, MM still represents an unmet medical need.

**Figure 1:**
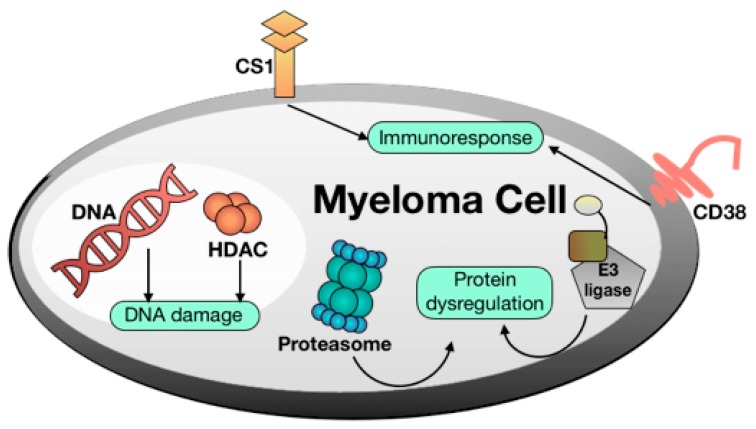
Molecular targets of multiple myeloma drugs.

During the last five years, new targets in MM have been proposed as therapeutic options (Figure 1), thus broadening the MM therapeutic landscape. Several drugs are now available and new molecules have recently been approved (Table 1) other therapies, including a peptide-based vaccination, are under validation [6]. Briefly, the main MM therapies consist of:

- DNA damaging agents. Alkylating agents such as melphalan and other DNA damaging agents such as doxorubicin as well as pan-HDAC inhibitors such as panobinostat are still important MM drugs [7].
- Proteasome inhibitors [8]. The proteasome is a multicatalytic target responsible for the degradation of 80–90% of proteins during cell life. Until now, three proteasome inhibitors have been approved for clinical use: Bortezomib, Carfilzomib, and Ixazomib. Particularly, Bortezomib was the first FDA-approved proteasome inhibitor and represents one of the most important discoveries for fighting MM of recent years. Of note, these agents can stimulate bone formation in MM patients [9]. Proteasome modulation can be also achieved by employing small molecules such as lenalidomide, structurally related to thalidomide and acting as E3-Ligasi inhibitors [10].
- Immunomodulators [11]. MM was among the first tumors wherein the therapeutic efficacy of blockade of inhibitory immune receptors, particularly the PD-1 axis, was demonstrated in preclinical models.
- Monoclonal antibodies [12]. There are only two FDA-approved monoclonal antibodies for the treatment of MM: daratumumab, targeting the CD38 pathway, and elotuzumab, targeting the SLAMF7 pathway. Despite the efficacy of this strategy in long term cancer remission, monoclonal antibodies still remain very expensive, thus limiting their diffusion.

It has to be pointed out that treatments currently available are often unsuccessful, lead to drug resistance [13], and cause adverse effects [14]. One of the major side effects related to MM therapies is a dose-limiting neurotoxicity, often linked to chemotherapy-induced peripheral neuropathy, which generally presents several sensory symptoms, including hypersensitivity, dysesthesia, and paresthesia [15,16]. At least 60% of patients treated with Bortezomib shows this severe side effect, leading to dose reduction or complete suspension of the therapy. 

Therefore, despite the efforts of the scientific community, MM therapy is still considered an unmet medical need and new effective therapies are needed.

Our research group has been studying MM from a pharmacological point of view for several years [17,18,19] and only recently we have focused on the identification of new therapeutic opportunities. Following a nature-aided drug discovery approach, we demonstrated the beneficial effects of *Hibiscus sabdariffa* Linn against MM [20]. *H. sabdariffa* is an annual herbaceous subshrub belonging to the Malvaceae family that is also known as Roselle, Red Sorrel or Karkadè. This plant is commonly distributed in tropical and subtropical regions and it is originally native to India and Saudi Arabia. It is cultivated in different regions such as Sudan, Egypt, Nigeria, Mexico, Saudi Arabia, Taiwan, West Indies, Central America, and European countries [21,22,23]. *H. sabdariffa* calyces are commonly used for the preparation of tea and infusions. The red drink is widely consumed directly or it is an ingredient in the preparation of some foods, such as juice, jam, pudding, gelatin, and desserts [24]. Moreover, it is well known in traditional medicine for its diuretic and mild laxative effects. Along the years, the phytochemical composition *H. sabdariffa* calyces as well as their pharmacological properties have been extensively studied. Different *H. sabdariffa* extracts have been prepared and the main constituents are phenols, polyphenols, anthocyanins, and organic acids, such as citric, tartaric, malic, ascorbic acids, and others [24,25]. Several studies demonstrated that *H. sabdariffa* calyces possess high antioxidant and anti-inflammatory properties and are able to counteract hypertension [26], diabetic status [27], microbial infections [28], as well as to slow down the development of atherosclerosis [29]. 

*H. sabdariffa* is known to possess antiproliferative properties and to be a possible source of new bioactive compounds for cancer treatment; it shows an antiproliferative effect against MCF-7 and MDA-MB-231 breast carcinoma cells, CaoV-3 ovarian cancer cells, Hela cervical cancer cells, and K-562 leukemia cells [30,31,32,33]. Moreover, anthocyanins extracted from *H. sabdariffa* are able to induce apoptosis of promyelocytic leukemia HL-60 cells via the p38 MAP kinase pathway [34].

To continue the study on the anticancer potential of *H. sabdariffa* calyces, specially focusing on MM, in this work we focused on the preparation and bioassay-guided fractionation of its ethanolic extract. Herein we report the isolation, structure elucidation, and evaluation of the potential of Hibiscus acid dimethyl ester and of a new aldehydic compound against multiple myeloma. In detail, their cytotoxicity and effect on cell migration and invasion of RPMI-8226 MM cells as well as their neurotoxicity on embryo rats dorsal root were evaluated.

## 2. Results and discussion

The dried and powdered *H. sabdariffa* calyces were extracted following a microwave-assisted solvent extraction (MASE) procedure exploiting 80% ethanol as the extracting solvent [35,36,37,38]. The obtained ethanolic extract (HsEE) was then fractionated via a liquid/liquid extraction, suspending the extract in water and sequentially extracting with hexane (A), dichloromethane (B), ethyl acetate (C), and butanol (D), yielding four organic fractions (HsFA–D) and an aqueous one.

**Figure 2:**
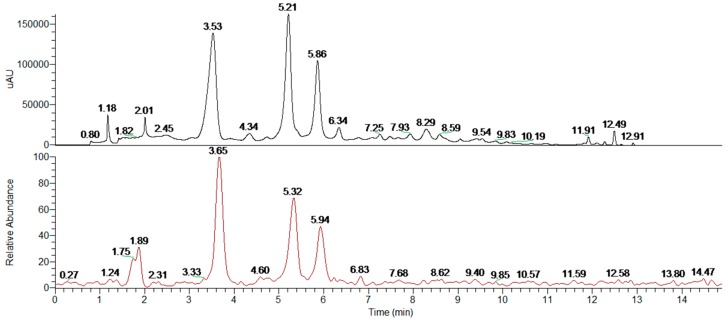
HPLC-UV/PAD-ESI/MS profile recorded at λ = 325 nm (black line) and reconstructed ion chromatograms (red line) of *H. sabdariffa* ethanolic extract (HsEE).

To obtain the analytical fingerprint, useful both as a control of the extracts’ quality and as a starting point for future purification studies, we set up a proper RP-HPLC-UV/PAD-ESI/MS method [39]. Specifically, different elution conditions (methanol, acetonitrile, water, with or without formic acid) adopting several gradient modes were tested on two different columns (Symmetry C18 and Chromolith Performance RP-18 endcapped). The best results in terms of peak resolution, peak shape, and time of analysis were achieved using a Symmetry C18 (5 µm, 3.9 × 150 mm) column in gradient elution conditions, using water and ACN added with formic acid. The HsEE HPLC-UV chromatograms (λ = 325 nm) together with the reconstructed ion chromatograms (RIC) in negative ion mode are shown in Figure 2. 

With the HPLC-UV-ESI/MS analytical methodology in hands, the profiles of fractions A–D were drawn: HsFA and HsFB contain only compounds with low polarity (retention time >12 min), HsFC presents acidic components (hibiscus acid, 5-caffeoylquinic acid and isomers, caffeic acid), while HsFD shows only few peaks at low intensity. The chromatographic profiles of HsFA–D and the MS associated with the peaks (total ion content) are reported in the Supplementary Material (Figure S1, Table S1). 

In the meantime, the effect of HsEE and HsFA-D on cell viability of RPMI 8226 cells was assessed by MTT vital count assay. Cells were treated with different concentrations of HsEE (1–50 mg/mL) or HsFA-D (1–10 mg/mL) for 24, 48, and 72 h. Untreated cells were used as control.

**Figure 3:**
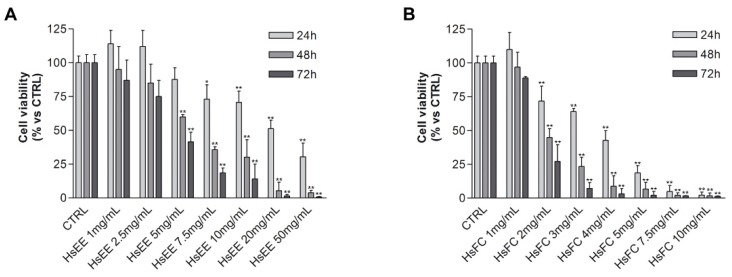
RPMI 8226 cell viability after HsEE or HsFC treatment. (**A**) MTT assay of RPMI 8226 cells treated with different concentrations (1–50 mg/mL) of HsEE for 24, 48, and 72 h. (**B**) MTT assay of RPMI 8226 cells treated with different concentrations (1–10 mg/mL) of HsFC for 24, 48, and 72 h. Untreated cells (CTRL) are control. Graphs represent the mean percentage ± SD of viable cells of three independent experiments (* *p* < 0.05, ** *p* < 0.01 vs. CTRL).

HsEE and HsFC are able to impair cell viability of RPMI 8226 cells in a dose- and time-dependent manner with IC_50_ at 24 h of 21.3 mg/mL and 3.5 mg/mL for HsEE and HsFC, respectively (Figure 3). The other fractions were not active and therefore were discarded (data not shown).

**Figure 4:**
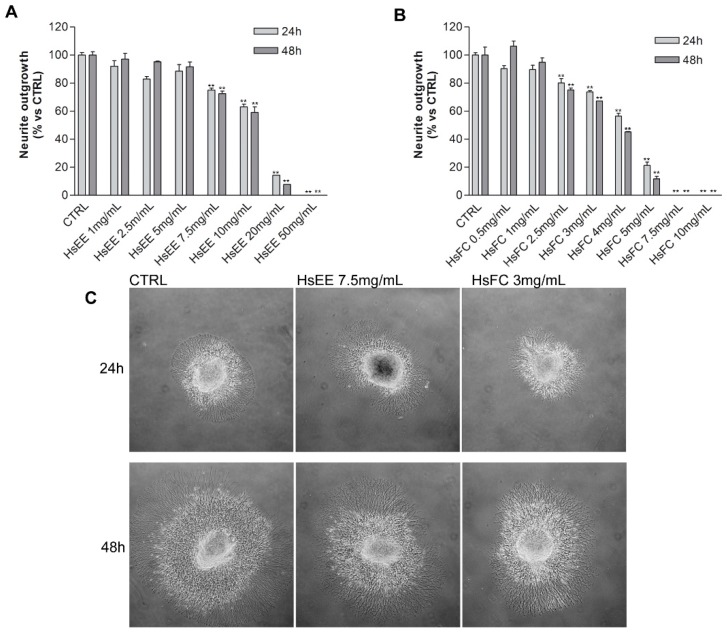
In vitro DRG neurotoxicity of HsEE and HsFC. (**A**) Neurite outgrowth from DRG treated with HsEE for 24 and 48 h. (**B**) Neurite outgrowth from DRG treated with HsFC for 24 and 48 h. Graphs represent mean neurite length ± SD of three independent experiments. (** *p* < 0.01 vs. CTRL). (**C**) Representative images of DRG treated with HsEE or HsFC for 24 and 48 h. Untreated DRG represent the controls.

The in vitro neurotoxicity of the crude extract, HsEE, and of the most effective fraction, HsFC, was then assessed by the well consolidated embryo rats dorsal root ganglia (DRG) neurotoxicity model [40]. Briefly, DRG explants from E15 Sprague Dawley rat embryos are able to sprout neurites when cultured with prodifferentiation agents, and the neurite outgrowth is blocked or slowed by neurotoxic agents. DRG were treated with different concentrations of HsEE (1–50 mg/mL) or HsFC (1–10 mg/mL) for 24 and 48 h and then the neurite outgrowth was measured. Results showed that HsEE and HsFC are non-neurotoxic at doses lower than 7.5 mg/mL and 3 mg/mL, respectively, after both 24 h and 48 h of treatment (Figure 4). At these time points, neurotoxicity was evident for both extracts at higher concentrations. HsEE is highly neurotoxic at its active concentrations (IC_50_ = 21.3 mg/mL), while HsFC can be considered safe at concentrations near its IC_50_ (IC_50_ = 3.5 mg/mL). 

We decided to further investigate HsFC at the effective and non-neurotoxic concentration of 3 mg/mL, evaluating the cell viability/cell death (RPMI 8226 cells) by Trypan blue vital count assay and the cell migration and invasion properties by Boyden chamber assay. 

**Figure 5:**
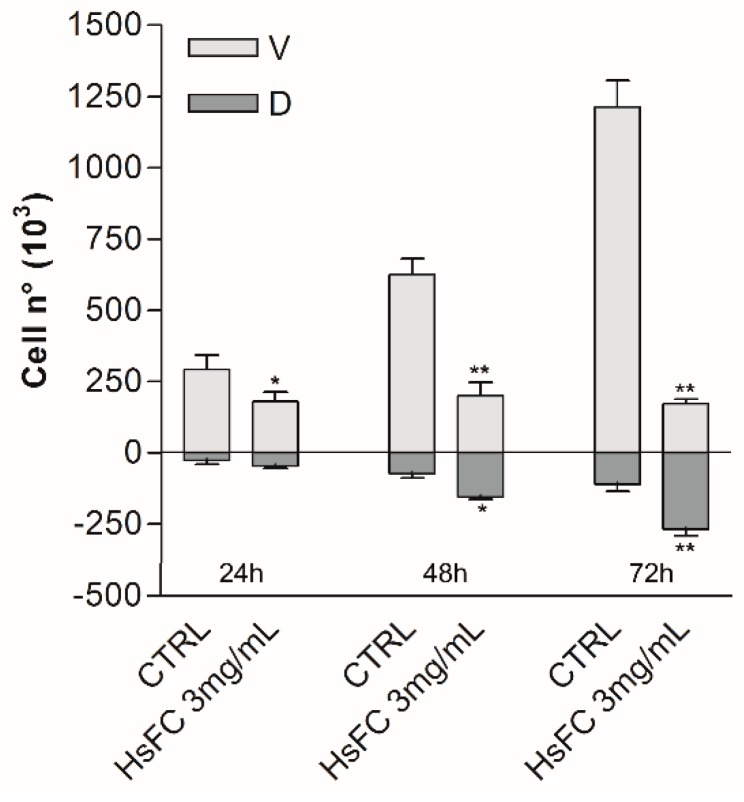
Trypan blue vital count assay of RPMI 8226 cells treated with HsFC 3 mg/mL. Untreated cells (CTRL) are control. Graphs represent the mean ± SD number of counted viable (V) and dead (D) cells of three independent experiments. (** *p* < 0.01 vs. CTRL).

**Figure 6:**
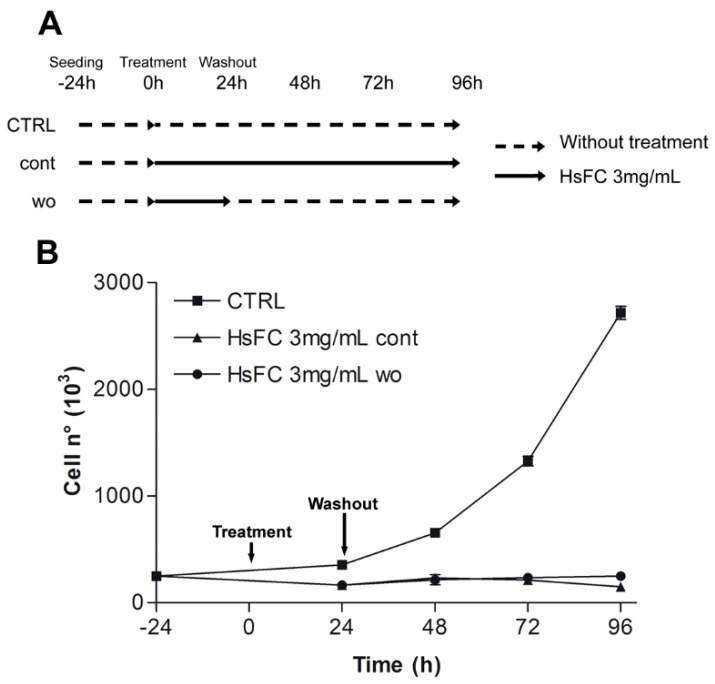
Effect of RPMI 8226 cells treated with HsFC 3 mg/mL in continuous (cont) or in washout (wo) culture medium. (**A**) Time schedule of HsFC treatment. Black arrows represent the presence of HsFC in culture medium of cont or wo treatment. Dashed lines represent culture medium without treatment. (**B**) Graphs represent the mean ± SD number of viable cells counted in three independent experiments.

**Figure 7:**
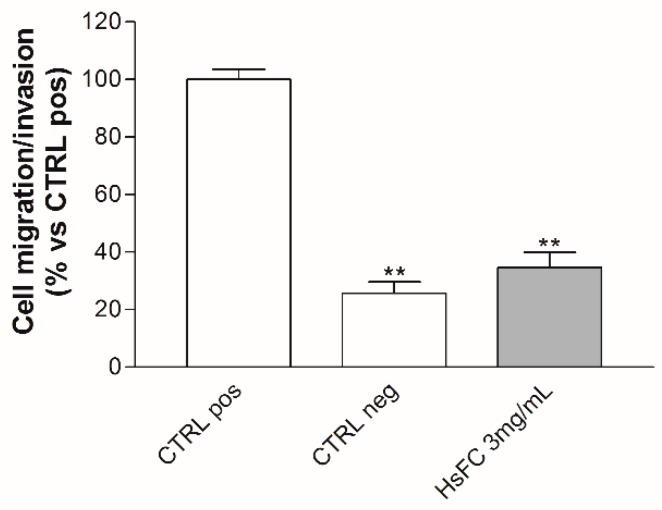
RPMI 8226 cell migration/invasion after HsFC treatment. The migration/invasion ability of RPMI 8226 cells was assessed by Boyden chamber assay. Cells were treated with HsFC 3 mg/mL. Untreated cells with or without FBS as chemoattractant represent positive controls (CTRL pos) and negative controls (CTRL neg), respectively. Data represent the percentage of migrating cells with respect to CTRL pos, arbitrarily set to 100%, and are expressed as mean ± SD of three independent experiments. (** *p* < 0.01 vs. CTRL pos).

Results of Trypan blue assay after treatment of RPMI 8226 cells with HsFC (3 mg/mL for 24, 48 and 72 h) highlighted that this fraction reduced cell viability and increased the number of dead cells from 8% (of untreated cells) to 20% (at 24 h), 43% (at 48 h), and 61% (at 72 h) (Figure 5). Moreover, the replacing of the culture medium after 24 h of treatment with fresh medium without HsFC generated a growing curve comparable to that obtained with continuous treatment; therefore, the HsFC effect is not reversible. (Figure 6). Regarding the cell migration and invasion, assessed by Boyden chamber assay, the treatment with HsFC significantly inhibited the ability of untreated RPMI 8226 cells in the presence of FBS as chemoattractant to pass through the gelatin-coated membrane. The HsFC effect was similar to that observed when untreated RPMI 8226 cells are in the absence of the chemoattractant FBS (Figure 7). Seventy percent of patients presents bone metastasis at diagnosis of MM and the percentage further increases during the development of the disease and can reach 90% of patients. For these reasons, it is important to identify drugs that are not only toxic to MM but that are also effective against MM bone metastasis formation.

To identify the main metabolites responsible for the cytotoxic properties, HsFC was then further fractionated. A first purification with polymer-supported carbonate (PS-carbonate) of HsFC was performed. Briefly, the fraction dissolved in methanol was treated with PS-carbonate, the solvent removed, and the resin washed with fresh methanol and dichloromethane. The washing solvent was collected and evaporated under reduced pressure, obtaining the wash fraction (WF). The PS-carbonate was then treated with 0.1% HCl in methanol, thus obtaining a simplified fraction. The subsequent purification by flash chromatography allowed the isolation of six fractions which underwent a preliminary cytotoxicity screening (MTT test). Only one fraction was active against MM in the initial screening. The HPLC-MS analysis showed that the white solid obtained corresponded to a pure compound with *m/z* 218. 

**Figure 8:**
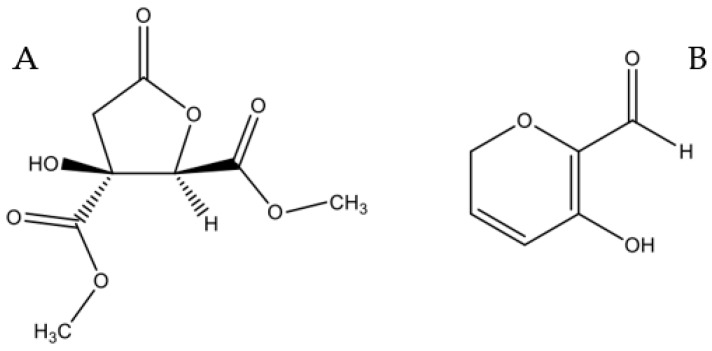
Chemical structure of *Hib-ester* (**A**) and *Hib-carbaldehyde* (**B**).

The ^1^H-NMR spectrum recorded in DMSO is consistent with that reported in literature for hibiscus acid dimethylester [41]. To confirm the γ-lactonic structure of this compound, bidimensional NMR (^1^H-^13^C HSQC *J* = 8 Hz, HSQC *J* = 2 Hz and ^1^H-^13^C HMBC) in CDCl_3_ was recorded (Figure S8). Basing on this data and on the positive value of its specific optical rotatory power recorded in chloroform and acetonitrile ([α]^D^_20_ = + 57.0 and + 84,1, respectively), the compound has been identified as (2S,3R)-hibiscus acid dimethylester (Figure 8A), by now on called *Hib-ester* [41].

It has to be noted that, according to the purification procedure, PS-carbonate caught acid compounds present in HsFC, including the hibiscus acid. 

Further fractionation of WF by flash chromatography yielded four main fractions, which underwent MTT cytotoxicity screening. A yellow oil, corresponding to new pure compound showing an interesting MTT value, was isolated. This compound was identified by NMR techniques (^1^H-NMR, ^1^H-^13^C HSQC *J* = 8 Hz, HSQC *J* = 2 Hz and 1H-13C HMBC) and MS analysis (*m/z* 126) as 5-hydroxy-2*H*-pyran-6-carbaldehyde, hereafter called *Hib-carbaldehyde* (Figure 8B). This compound has not been isolated from natural sources before. 

The effect of *Hib-ester* and *Hib-carbaldehyde* on cell viability of RPMI 8226 cells was evaluated by MTT and trypan blue vital count assay. Cells were treated with increasing concentrations of the compounds (3 µg/mL–3 mg/mL) for 24, 48, and 72 h and untreated cells were used as control.

**Figure 9:**
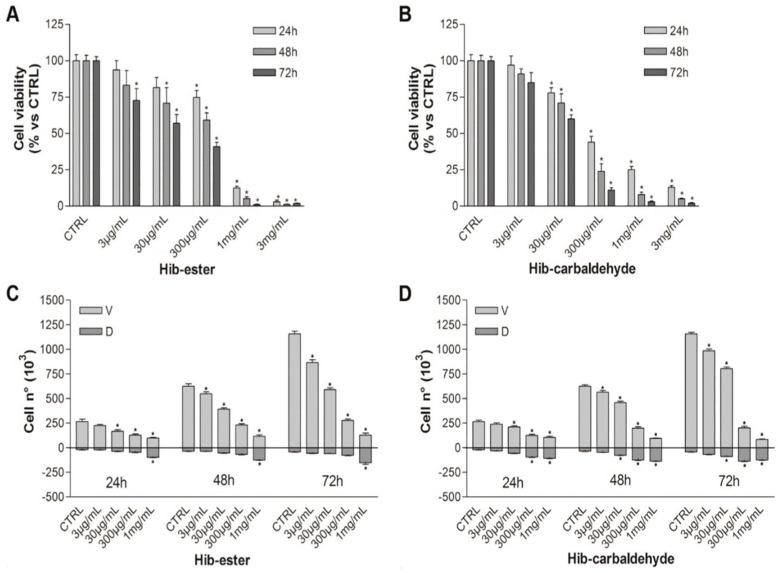
Cell viability and neurotoxicity after treatment with *Hib-ester* and *Hib-carbaldehyde*. (**A**) MTT assay of RPMI 8226 cells treated with *Hib-ester* (3 µg/mL–3 mg/mL) for 24, 48, and 72 h. (**B**) MTT assay of RPMI 8226 cells treated with *Hib-carbaldehyde* (3 µg/mL–3 mg/mL) for 24, 48, and 72 h. (**C**) Trypan blue vital count assay of RPMI 8226 cells treated with *Hib-ester* (3 µg/mL–1 mg/mL) for 24, 48, and 72 h. **D**) Trypan blue vital count assay of RPMI 8226 cells treated with different concentrations of *Hib-carbaldehyde* (3 µg/mL–1 mg/mL) for 24, 48, and 72 h.

Both compounds significantly reduced cell viability of RPMI 8226 cells (MTT test) in a dose- and time-dependent manner (Figure 9A,B). After 24 h of treatment, *Hib-ester* and *Hib-carbaldehyde* are about ten and 20 times more active than HsFC, showing IC_50_ values of 0.45 and 0.21 mg/mL, respectively. Results of the trypan blue vital count assay highlighted a similar trend (Figure 9C,D).

The evaluation of the in vitro neurotoxicity of *Hib-ester* and *Hib-carbaldehyde* using our DRG model showed that both compounds are non-neurotoxic at doses near the IC_50_ values, after both 24 h and 48 h of treatment. *Hib-carbaldehyde* at 0.3 mg/mL and 1 mg/mL reduced the length of neurites, but at a non-neurotoxic percentage (about 20%) (Figure S8).

**Figure 10:**
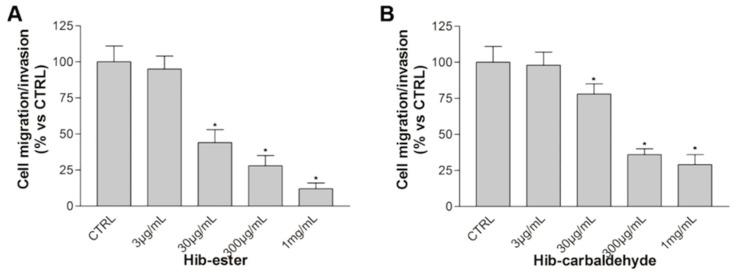
RPMI 8226 cells’ migration/invasion after *Hib-ester* and *Hib-carbaldehyde* treatment. Migration/invasion ability of RPMI 8226 cells was assessed by Boyden chamber assay. (**A**) Cells were treated with *Hib-ester* (3 µg/mL–1 mg/mL). (**B**) Cells were treated with *Hib-carbaldehyde* (3 µg/mL–1 mg/mL). Data represent the percentage of migrating cells with respect to CTRL, arbitrarily set to 100%, and are expressed as mean ± SD of three independent experiments. (** *p* < 0.01 vs. CTRL pos).

Regarding the ability to impair cell migration and invasion, both compounds are effective at their respective IC_50_ values. Particularly, *Hib-ester* inhibits cell migration and invasion by more than 50% at 30 µg/mL (Figure 10A,B). 

## 3. Conclusions

In this paper, we prepared a hydroalcoholic *H. sabdariffa* extract by adopting a MASE-based methodology, an efficient method to extract secondary metabolites from raw matrices with high yields and in short time. Microwave oven parameters (temperature, power, and time) were chosen according to literature data concerning the stability of the main metabolites of *H. sabdariffa* [42]. The bioguided fractionation of the crude ethanolic extract (HsEE) (sequential liquid/liquid extraction using organic solvents with growing polarities, followed by chromatographic fractionation) allowed the identification of two active compounds, named *Hib-ester* and *Hib-carbaldehyde* (Figure 8).

Both compounds are effective against the tested MM cell line at non-neurotoxic µg/mL concentrations. Moreover, they are able to reduce cell migration and invasion, events involved in the process of metastasis. Compounds identified in this study may be the starting point for the antineoplastic medicinal chemistry field. We expect that our results can offer useful information for the identification of next-generation drugs against multiple myeloma.

## 4. Materials and Methods 

### 4.1. Chemicals and Standards

Solvents for both HPLC (HPLC grade) and extraction procedures (analytical grade) were supplied by Carlo Erba (Milan, Italy). Chlorogenic acid was purchased from Indena S.p.A. (Milan, Italy).

Analytical thin-layer chromatography (TLC) was carried out on silica gel precoated glass-backed plates (Fluka Kieselgel 60 F254, Merck, Darmstadt, Germany) and visualized by UV light or ceric ammonium molibdate (IV) stain (Hanessian’s Stain). 

Flash chromatography was performed with silica gel 60 (particle size 230–400 mesh) purchased from Nova Chimica (Cinisello Balsamo, Italy).

Deuterated solvent for NMR spectroscopy, RPMI-1640 Medium, Phosphate Buffered Saline (PBS), 3-(4,5-dimethylthiazol-2-yl)-2,5-diphenyltetrazolium bromide (MTT), dimethylsulfoxide (DMSO) were purchased from Sigma Aldrich (Milan, Italy).

Fetal bovine serum (FBS; GibcoR) was purchased from ThermoFisher Scientific (Lisbon, Portugal).

### 4.2. Instruments

*H. sabdariffa* calyces were powdered using a blade mill (A10 IKA-Werke GmbH & Co., Staufen, Germany) and then extracted exploiting a Multimode Microwave apparatus (MARSX press, CEM Corporation, Matthews, NC, USA).

All solvents were evaporated under reduced pressure using a Heidolph Laborota 4000 instrument (Heidolph Instruments GmbH & Co., Schwabach, Germany). 

^1^H-NMR, ^13^C-NMR, and bidimensional experiments were performed at 400.0 MHz and 100.6 MHz, respectively, on the Bruker Avance 400 MHz FT NMR spectrometer (Bruker, Leipzig, Germany) with a multinuclear BBO probe. CDCl_3_ was used as solvent. 1H chemical shift values were reported on the δ scale in ppm, relative to TMS (δ = 0.0 ppm) and in ^13^C-NMR, chemical shift values were reported posing CDCl3 (δ = 77.36 ppm) as reference. 

High performance liquid chromatography-electrospray-tandem mass spectrometry (RP-HPLC-UV/PAD-ESI/MS) analyses were carried out on Finnigan LCQ fleet ion trap system, controlled by Xcalibur software 1.4 (ThermoFinnigan, San Jose, CA, USA).

### 4.3. Plant Material and Extraction Procedure

*H. sabdariffa* plants obtained from seeds were cultivated in pots under greenhouse conditions (mean temperature 25.9 °C) at CREA institute (43°49′05″N, 7°45′30″E, Sanremo, IM, Italy).

*H. sabdariffa* calyces from five-month-old plants were harvested at the end of September, separated from the seedpod, and after a rapid wash with tap water, dried in a ventilated oven at 45 °C for three days. Voucher samples of dry calyces (ca. 100 g) are conserved under room conditions at the Plant Propagation Laboratory of the CREA, Sanremo, Italy. Seeds of this HS line are also kept for regular use.

The obtained matrix was stored in dark conditions and, at the time of use, it was cut to small size and grounded with a blade mill. Then, 10 g of the obtained homogeneous fine powder were dispersed in 200 mL of ethanol 80% and subjected to microwave heating (2 min ramping, 5 min hold time, maximum pressure 120 psi, maximum potency 200 W, temperature 50 °C, one cycle). The mixture was left to cool at room temperature filtrated and concentrated under reduced pressure to obtain HsEE as a red thick oil.

### 4.4. Extract Fractionation and Analysis

The crude extract HEE was resolubilized in water and sequentially extracted three times. with hexane (A), dichloromethane (B), ethyl acetate (C), and butanol (D) as organic solvents. The combined organic phases were dried with anhydrous NaSO_4_ and the solvent removed under reduced pressure, yielding four organic fractions (HsFA–D) and an aqueous fraction. All dry extracts obtained were stored at 4 °C until analysis. Each extraction experiment was carried out in triplicate. HsEE and HsFA–D were analyzed using high-performance liquid chromatography-electrospray-tandem mass spectrometry. Each sample was dissolved in water (4 mg/mL) and filtered with a 0.45 μm GH Polypro (GHP) membrane before injection into the HPLC system. The sample was analyzed on a Symmetry C18 (5 µm, 3.9 × 150 mm) column. The mobile phase is composed by Water (A) and ACN (B), both containing 0.1% (*v*/*v*) formic acid and the composition gradient was: an initial isocratic elution of 10% B for 2min, from 10 to 25% B in 8 min, from 25 to 90% B in 5 min, 10% B until 3 min, followed by a re-equilibration step of 4 min. The flow rate was 1 mL/min and the elution was performed at room temperature. The UV detection was fixed at 325 nm. Mass spectra were generated in negative ion mode under constant instrumental conditions (ion spray voltage 5 kV, capillary voltage −12 V, capillary temperature 220 °C, and tube lens voltage −60 V). MS/MS data were acquired in Dependent scan mode (Full-scan MS followed by MS/MS of the most intense ion).

HsFC (2.5 g) was dissolved in 50 mL of methanol and then subjected to a treatment with PS-carbonate (5 g). The mixture was shaken at room temperature for 40 min, then filtered and washed with methanol (30 mL) and dichloromethane (150 mL). The combined filtrates were evaporated under reduced pressure. The resin was then recovered and 50 mL of MeOH + 0.1% HCl was added. The system was shaken for 1 h at room temperature, and the solvent filtered. The procedure was performed twice. The combined methanolic filtrates were evaporated under reduced pressure and then subjected to a flash chromatography on silica gel with 50% Toluene, 30% hexane, and 20% isopropyl-alcohol as mobile phase. Six fractions were collected on the basis of the TLC profile. Fraction 2 (18 mg) corresponds to a single spot (TLC: MP = 5 Toluene: 3 hexane: 2 isopropyl-alcohol RF = 0.41). It underwent Nuclear Magnetic Resonance (1H- and 13C-NMR) and Mass Spectral (MS) analysis and it has been identified as dimethyl 3-hydroxy-5-oxotetrahydrofuran-2,3-dicarboxylate. All the spectra are reported in supplementary material, Figure S2. ESI-MS: *m/z* 218 [M + H]+. 1H-NMR (CDCl_3_, 400 MHz): δH: 5.1 (1H, s), 3.9 (3H, s), 3.8 (3H, s), 3.0 (1H, d, *J* = 17.4 Hz), 2.8 (1H, d, *J* = 17.4 Hz). 13C-NMR (CDCl_3_): δC: 172, 168, 165, 82, 77, 55, 54, 40. 

HsFC (5 g) was dissolved in 100 mL of methanol and then subjected to a treatment with PS-carbonate (10 g). The mixture was shaken at room temperature for 40 min, then filtered and washed with dichloromethane (300 mL). The filtrate was concentrated in vacuo and then subjected to a flash chromatography on silica gel with 70% dichloromethane and 30% ethyl acetate as mobile phase. Four fractions were collected on the basis of the TLC profile. The second fraction corresponds to a pure compound (TLC: MP = 7 DCM: 3 AcOEt RF = 0.37; 9 mg) as a yellow oil. Its structure was then incestigated with Nuclear Magnetic Resonance (^1^H- and ^13^C-NMR) and Mass Spectral (MS) techniques and elucidated as 5-hydroxy-2*H*-pyran-6-carbaldehyde (see supplementary material S2). ESI-MS: *m/z* 127 [M + H]+, 149 [M + Na]+. 1H-NMR (CDCl_3_, 400 MHz): δH: 9.6 (1H, s), 7.2 (1H, m), 6.4 (1H, m), 4.6 (2H, d).13C-NMR (CDCl_3_): δC: 178, 160, 155, 125, 110, 58.

### 4.5. Cell Culture 

RPMI 8226 human multiple myeloma cells (ATCC, Manassas, VA, USA) were cultured in RPMI 1640 medium (Euroclone, Pero, Italy) supplemented with 10% fetal bovine serum (FBS), 1% glutamine, 1% Penicillin and Streptomycin (Euroclone, Pero, Italy). Cells were incubated at 37 °C and 5% CO_2_ in a humidified incubator. 

HsEE and HsFC were dissolved in phosphate-buffered saline (PBS) at 1 g/mL concentration and then diluted directly into culture medium to working concentrations. 

### 4.6. Biological Assays

#### 4.6.1. MTT Assay

RPMI 8226 cells were seeded in 96-well plates at 10 × 10^3^ cells/well density and were treated with different concentrations of HsEE (1–50 mg/mL) and HsFC (1–10 mg/mL). Untreated cells represented the controls. After 24, 48, and 72 h of treatment, a 5 mg/mL solution of 3-(4,5-dimethylthiazol-2-yl)-2,5-diphenyltetrazolium bromide (MTT) (Sigma-Aldrich, St. Louis, MO, USA) was added directly to culture medium, at final concentration of 0.5 mg/mL. After 4 h of incubation at 37 °C, plates were centrifuged at 2 × 10^3^ RPM and culture medium was removed, formazan crystals were solubilized in acidified 2-propanol (HCl 0.3%), and absorbance was read at 560 nm in a microplate reader (BMG-Labtech, Offenburg, Germany).

#### 4.6.2. Trypan Blue Vital Count

RPMI 8226 cells were seeded in six-well plates at 250 × 10^3^ cells/well density and were treated with different concentrations of HsFC. Untreated cells represented the controls. After 24, 48, and 72 h of treatment cells were harvested and stained with Trypan blue vital dye (Sigma-Aldrich, USA). Viable and dead cells were counted in a Burker hemocytometer under a light microscope (Eclipse TS100, Nikon, Tokyo, Japan). 

The reversibility of HsFC was evaluated by Trypan blue vital count. Cells were plated and treated as previously described. After 24 h of treatment cells were harvested. Culture medium was removed after centrifugation at 1200 RPM and it was replaced with fresh medium without HsEE/HsFC. After 24, 48, and 72 h cells were stained and counted as previously described. 

#### 4.6.3. Boyden Chamber Assay

Boyden chamber assay was used to assess RPMI 8226 cell migration and invasion as previously described [20]. Briefly, 5 × 10^3^ RPMI 8226 cells were plated in HsEE/HsFC serum free medium in the upper compartment of the Boyden chamber. Culture medium supplemented with 10% FBS as chemo-attractant was placed in the lower compartment of the chamber. Compartments were separated by a polycarbonate membrane with 8 µm pores (Biomap, Agrate Brianza, MB, Italy). The membrane was coated with gelatin at 0.2 mg/mL concentration. 

After 16 h of incubation at 37 °C, the membrane was removed, fixed with cold methanol, and stained with Diff quick staining kit (Biomap, Agrate Brianza, MB, Italy). Cells on the lower surface of the membrane were counted under a light microscope. 

#### 4.6.4. DRG Neurotoxicity Assay

All experimental procedures were approved by the Ethics Committee for Animal Studies of the University of Milan Bicocca. 

A pregnant Sprague Dawley rat (Envigo, Udine, Italy) was sacrificed after a deep anesthesia. Embryos were collected in L15 medium (Euroclone, Pero, Italy) and DRG were removed after dissection. 

DRGs were seeded in 35 mm dishes previously coated with collagen and filled with AN2 medium (MEM (Euroclone, Pero, Italy), 10% Calf Bovine Serum (Euroclone, Pero, Italy), 1.4 mM L-Glutamine (Euroclone, Pero, Italy), 0.6% Glucose (Sigma-Aldrich, USA), and 5 ng/mL Nerve Growth Factor (NGF) (Thermo Fisher, Waltham, MA, USA). After 2 h, culture medium was removed and replaced with treatment medium (HsEE 1–10 mg/mL or HsFC 1–5 mg/mL). Untreated DRG represented the controls. After 24 and 48 h, micrographs of DRG were taken under a light microscope (Nikon Eclipse TS100) and the length of DRGs neurites was measured with ImageJ software (National Institutes of Health, Bethesda, MD, USA, http://imagej.nih.gov/ij).

### 4.7. Statistical Analysis 

Data are reported as mean ± standard deviation (SD) from at least three independent experiments. Statistical analysis was performed using GraphPad Prism 3 software. The differences between control and treated cells were evaluated using One Way ANOVA analysis of variance followed by Dunnet’s multiple comparison test. Statistical significance was set at *p* < 0.05 or *p* < 0.01.

## Figures and Tables

**Table 1:** Drugs approved for multiple myeloma and their molecular targets.

Drug	Trade Name	Target
Melphalan	Alkeran^®^	DNA
Bendamustine	Levact^®^, Ribomustin^®^, Treanda^®^	DNA
Doxorubicin	Adriamycin^®^	DNA
Carmustine	BiCNU^®^	DNA
Cyclophosphamide	Cytoxan^®^, Neosar^®^	DNA
Panobinostat	Faridak^®^	HDAC
Elotuzumab	Empliciti^®^	CS1
Daratumumab	Darzalex^®^	CD38
Bortezomib	Velcade^®^	Proteasome
Ixazomib	Ninlaro^®^	Proteasome
Carfilzomib	Kyprolis^®^	Proteasome
Thalidomide	Thalidomide Celgene^®^	E3 ligase
Lenalidomide	Revlimid^®^	E3 ligase
Pomalidomide	Imnovid^®^	E3 ligase

## Supplementary Materials

The following are available online at https://www.mdpi.com/1420-3049/24/13/2500/s1. Figure S1: HPLC-UV profile recorded of HsEE, HsF A, HsF B, HsF C and HsF. Table S1: Main peaks of HsEE and HsF A-D. Figure S2: MS/MS spectrum m/z293 Hibiscus acid Hibiscus acid 6-methyl ester and Caffeic acid derived. Figure S3: NMR spectra of Hib-ester. Figure S4: IR analysis of Hib-ester. Figure S5: MS analysis of Hib-ester. Figure S6: NMR spectra of Hib-carbaldehyde. Figure S7: MS analysis of Hib- carbaldehyde. Figure S8: Neurite outgrowth from DRG treated with Hib-ester and with Hib- carbaldehyde.
