# Supplementary material for: Anti-Multiple Myeloma Potential of Secondary Metabolites from Hibiscus sabdariffa
Source: Molecules. 2019 Jul 9;24(13):2500. doi: 10.3390/molecules24132500 (PMC6651714; doi:10.3390/molecules24132500)
Supplement: Supplementary file 1 [file molecules-24-02500-s001.pdf]

SUPPLEMENTARY MATERIAL

Anti-Multiple Myeloma Potential of Secondary Metabolites from *Hibiscus sabdariffa*

Alessio Malacrida [1,2,†], Valeria Cavalloro [3,†], Emanuela Martino [3,*], Arianna Cassetti [4], Gabriella Nicolini [1,2], Roberta Rigolio [1,2], Guido Cavaletti [1,2], Barbara Mannucci [5], Francesca Vasile [6], Marcello Di Giacomo [7], Simona Collina [7,*] and Mariarosaria Miloso [1,2,*]

[1] School of Medicine and Surgery, University of Milan-Bicocca, 20900 Monza, Italy; alessio.malacrida@unimib.it (A.M.); gabriella.nicolini@unimib.it (G.N.); roberta.rigolio@unimib.it (R.R.); guido.cavaletti@unimib.it (G.C.)
[2] Experimental Neurology Unit, University of Milano-Bicocca, 20900 Monza, Italy
[3] Department of Earth and Environmental Sciences, University of Pavia, 27100 Pavia, Italy; valeria.cavalloro01@universitadipavia.it
[4] CREA, Research Centre for Vegetable and Ornamental Crops, 18038 Sanremo, Italy; arianna.cassetti@CREA.gov.it
[5] Center of Large Equipment, University of Pavia, 27100 Pavia, Italy; barbara.mannucci@unipv.it
[6] Department of Chemistry, University of Milan, 20133 Milano, Italy; francesca.vasile@unimi.it
[7] Department of Drug Sciences, University of Pavia, 27100 Pavia, Italy; marcello.digiacomo@unipv.it
* Correspondence: emanuela.martino@unipv.it (E.M.); simona.collina@unipv.it (S.C.); mariarosaria.miloso@unimib.it (M.M.); Tel.: +39-0382-986810 (E.M.); +39-0382-987379 (S.C.); +39-0264-488123 (M.M.)
† Authors contributed equally to this work

S1. Chromatographic characterization of the extracts

Metabolites contents of HsEE and Fractions A-D were characterized *via* HPLC-UV/PAD-ESI-MS/MS analysis. Their chromatographic profiles and the MS associated to the peaks (total ion content) are below reported (Figure S1, Table S1). We also report the MS/MS spectra of the main peaks present in the chromatographic profile (Figure S2)

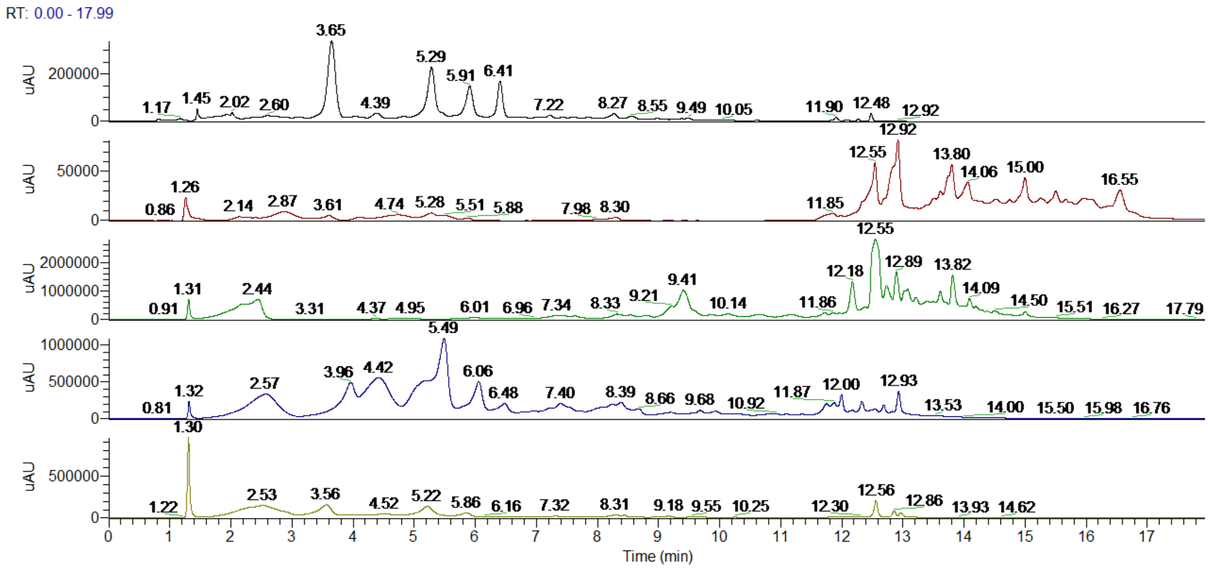

Figure S1. HPLC-UV profile recorded at λ = 325 nm of HsEE (black line), HsF A (red line), HsF B (green line), HsF C (blue line) and HsF D (yellow line).

Table S1. Main peaks of HsEE and HsF A-D.

	Rt (min)							
	**HsEE**	**A**	**B**	**C**	**D**	**m/z negative ion**	**m/z daughter ion**	**compound**
1	1,98	-	-	-	1.94	189	127	Hibiscus acid
2	2.60	-		2,57	2,53	203	185	Hibiscus acid 6-methyl ester
3	3,65			3,96	3,56	353	nd	3-Caffeoylquinic acid[b]
4	5,29			5,49	5,22	353	nd	5-CQA[a]
5	5,91			6,06		353	nd	4-Caffeoylquinic acid[b]
6	6,41			6,48		179	nd	Caffeic acid
7			9,41			293	179	Caffeic acid derived
8		12,18				312	nd	Unknown
9		12,55	12,55	12,55	12,56	312	nd	Unknown
10		12,92	12,89	12,93	12,86	393	nd	Unknown
11		13,80	13,82			669	nd	Unknown
12		15:00				371	nd	Unknown
13		15:50				721	nd	Dp-samb monoester[b]

[a]Confirmed with authentic standard. [b]Tentatively identified

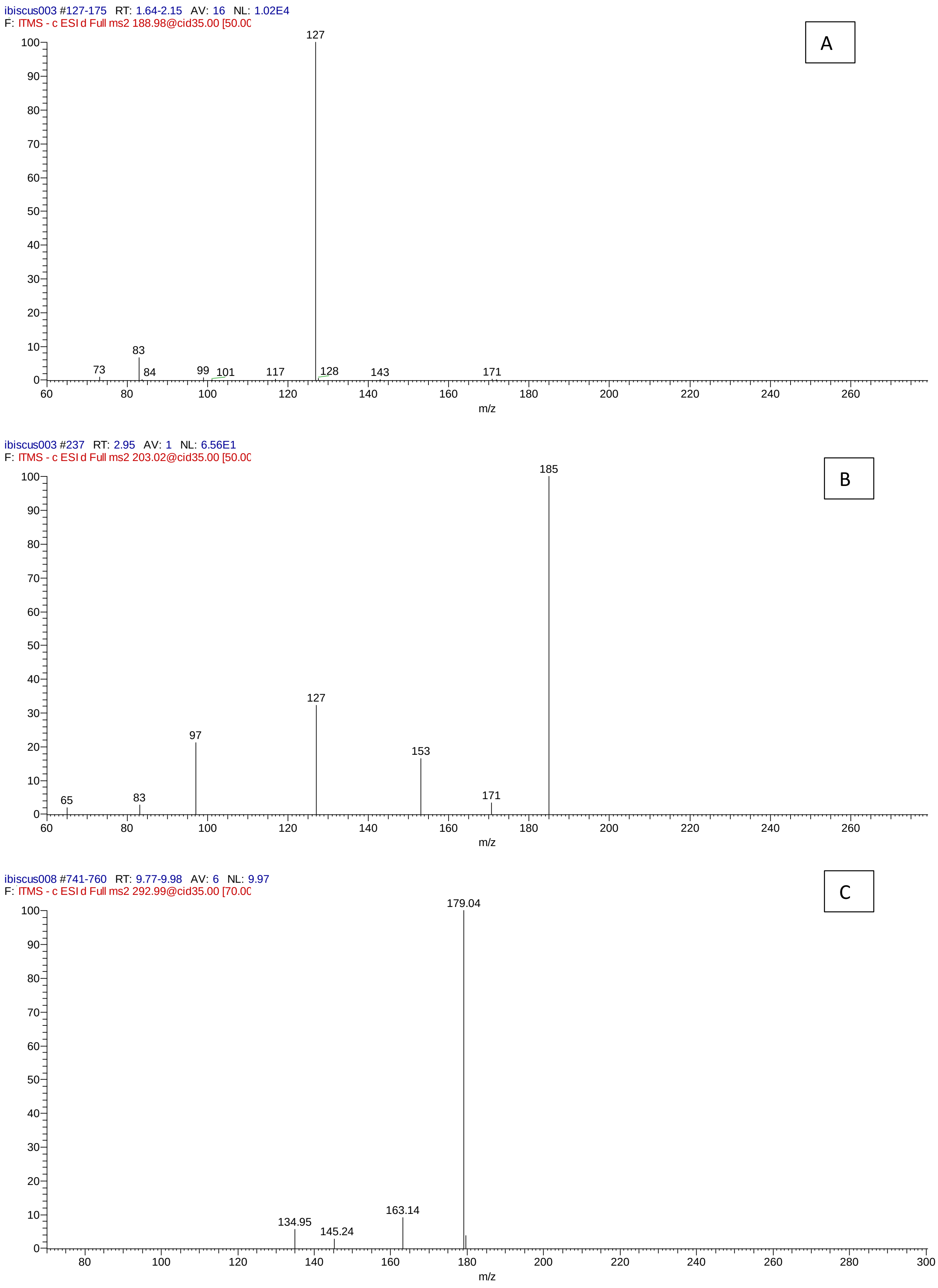

Figure S2: MS/MS spectrum m/z293 Hibiscus acid (Rt 1.98; A); Hibiscus acid 6-methyl ester (Rt 2.53; B); Caffeic acid derived (Rt 9.41; C)

S2. Secondary metabolites identification

Hibiscus acid dimethyl ester is a white solid, melting point: 124.3°C

- NMR analysis of hibiscus acid dimethyl ester:

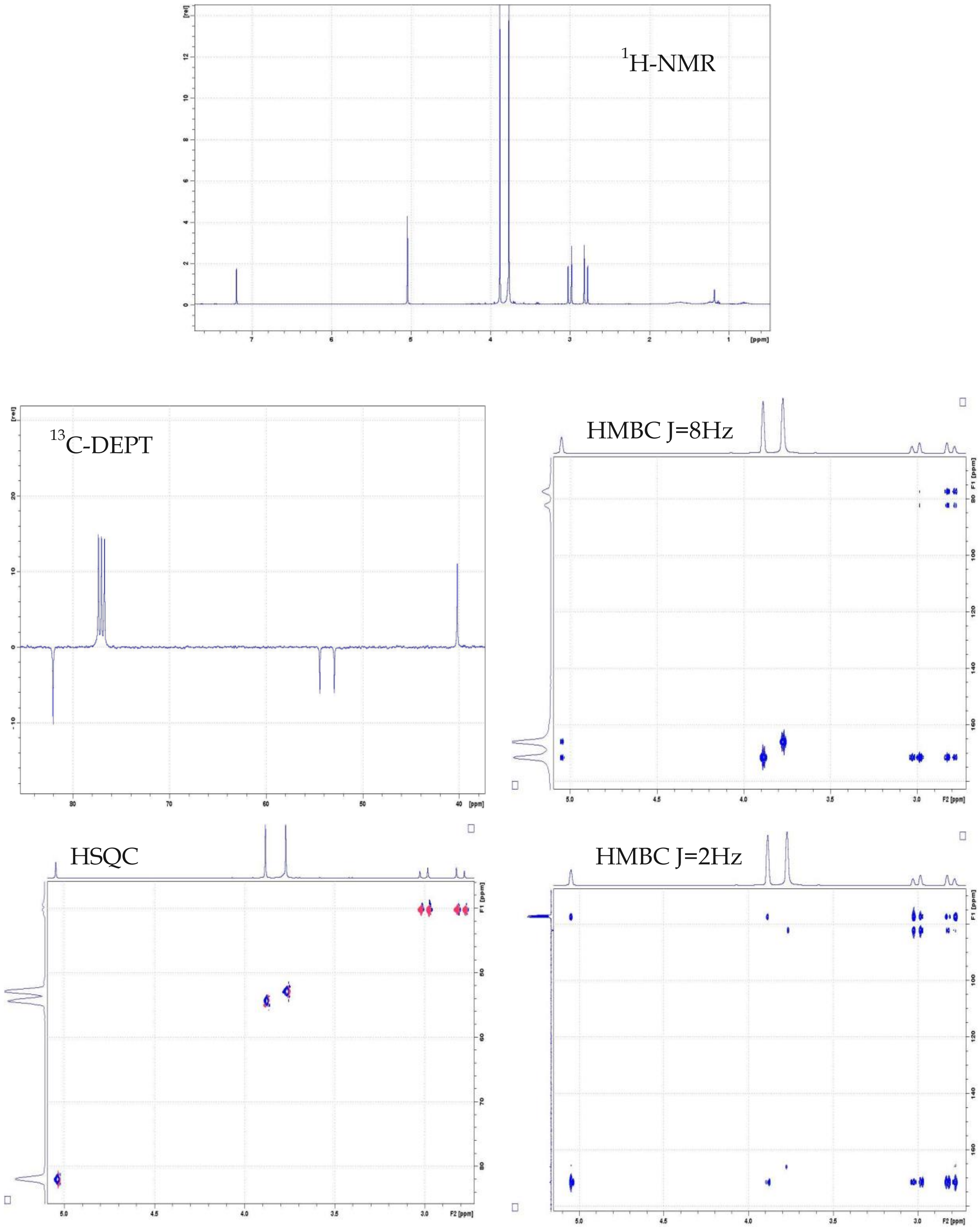

Figure S3. NMR analysis of *Hib-ester*

- IR analysis of hibiscus acid dimethyl ester:

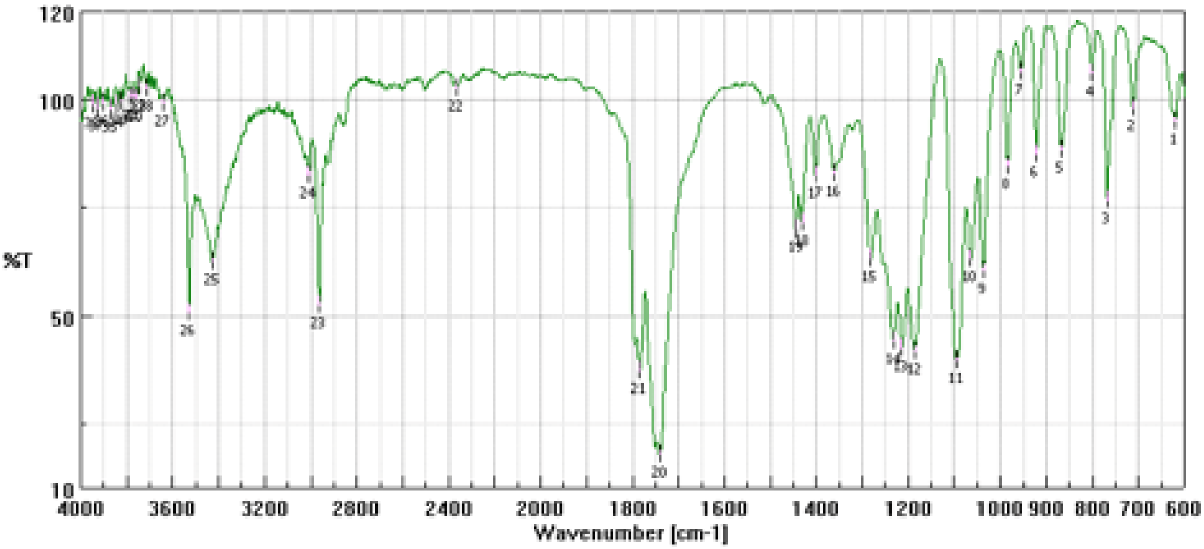

Figure S4. IR analysis of *Hib-ester*

Wavenumber [cm-1]	%T				
619.038	94.8529	1064.51	62.9806	1444.42	69.8627
711.604	97.882	1096.33	39.7184	1740.44	17.7719
767.53	76.5661	1186.97	41.8091	1783.83	37.0691
801.278	105.825	1213.97	42.5542	2363.34	102.538
866.846	88.2895	1233.25	44.2167	2963.09	52.2969
921.807	87.395	1282.43	63.1368	3008.41	82.3486
955.555	106.083	1361.5	82.86	3429.78	62.4903
984.482	84.3889	1402	82.2685	3529.09	50.8291
1036.55	60.291	1431.89	71.4603	3641.91	99.002

- MS analysis of hibiscus acid dimethyl ester:

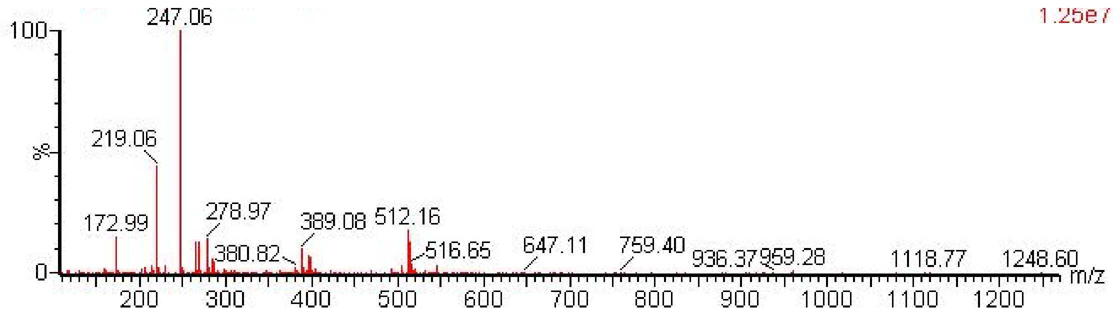

Figure S5. MS analysis of *Hib-ester*

5-hydroxy-2*H*-pyran-6-carbaldehyde (*Hib- carbaldehyde*) appears like a yellow oil.

- NMR analysis of 5-hydroxy-2*H*-pyran-6-carbaldehyde:

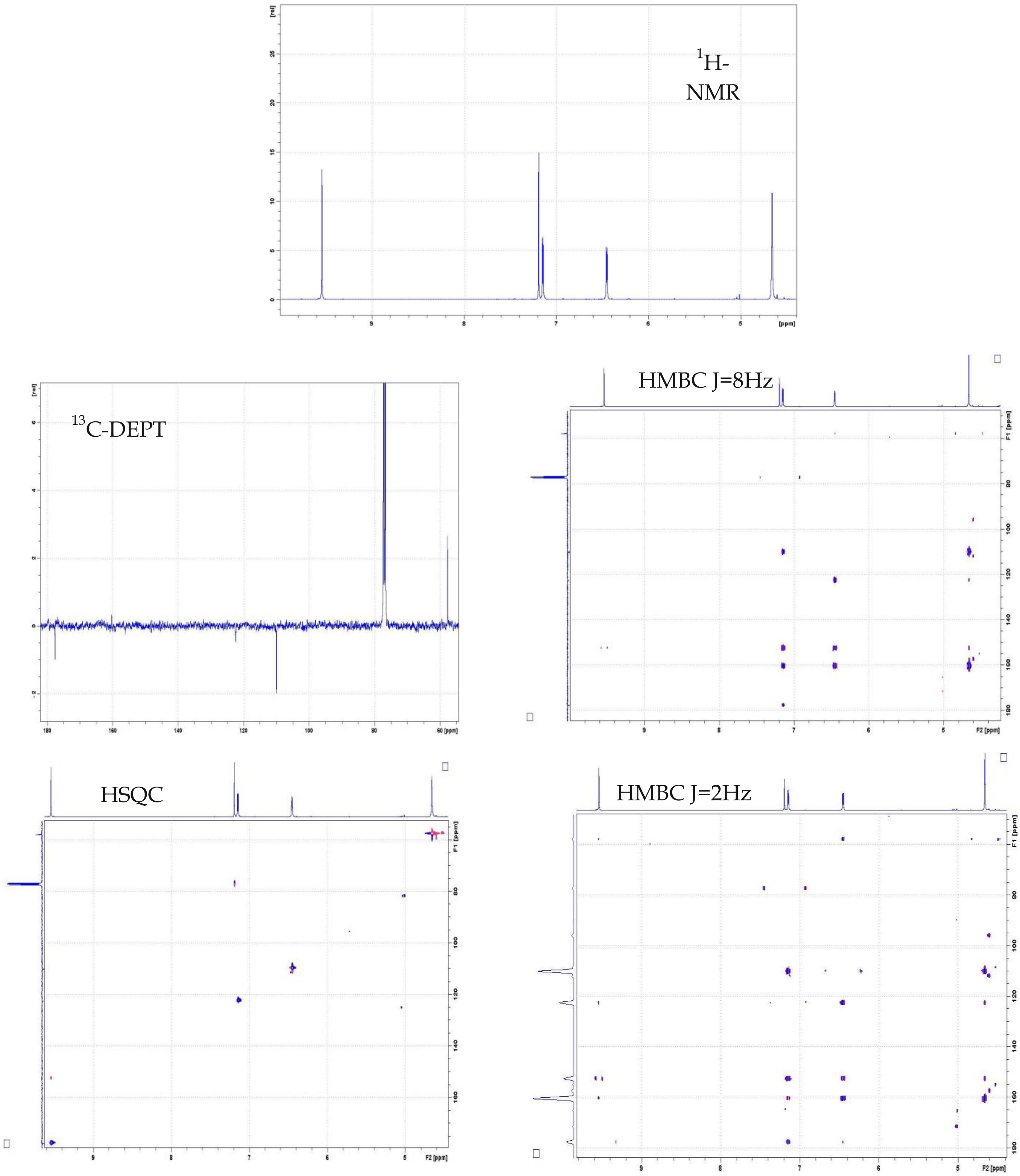

Figure S6. NMR analysis of *Hib-carbaldehyde*

- MS analysis of 5-hydroxy-2*H*-pyran-6-carbaldehyde:

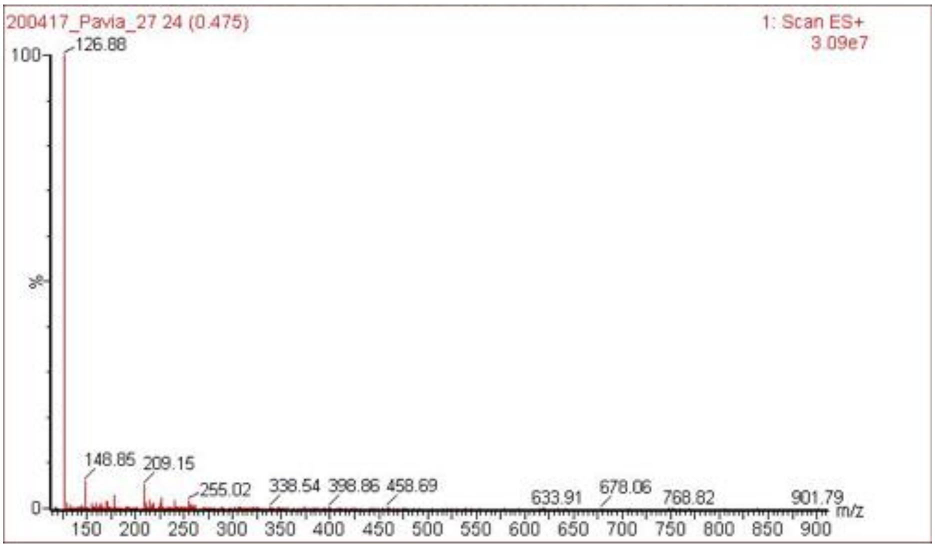

Figure S7 MS analysis of *Hib- carbaldehyde*

S3. Neurite outgrowth after treatment with the isolated *Hib- ester* and *Hib- carbaldehyde*

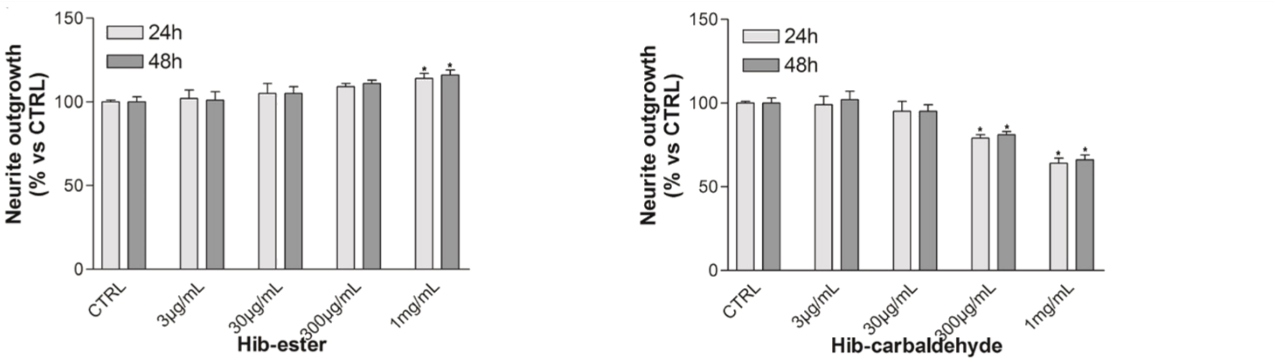

Figure S8 Neurite outgrowth from DRG treated with *Hib-ester* (3µg/ml – 1mg/ml) for 24 and 48h and with *Hib-carbaldehyde* (3µg/ml – 1mg/ml) for 24 and 48h.Untreated cells (CTRL) are control. Graph represent the mean ± SD of three independent experiments (* P<0.05, ** P<0.01 vs CTRL).
